# Multidimensional Symptom Burden Among Institutionalised Older Adults: Associations with Functional Status, Psychological Factors and Quality of Life

**DOI:** 10.3390/healthcare14183117

**Published:** 2026-09-21

**Authors:** Lucinda Marques, Lara Guedes de Pinho, Elisabete Alves, Isabel Bico, Manuel José Lopes

**Affiliations:** 1 Nursing Department, University of Évora, 7000-811 Évora, Portugal; 2 Comprehensive Health Research Centre (CHRC), 7000-811 Évora, Portugal; l.pinho@ess.ipvc.pt; 3 LA REAL—Associated Laboratory in Translation and Innovation Towards Global Health, University of Évora, 7000-811 Évora, Portugal; 4 Escola Superior de Saúde, Universidade Politécnica de Viana do Castelo, 4900-347 Viana do Castelo, Portugal; 5 Health Sciences Research Unit: Nursing (UICISA:E), Núcleo da Universidade Politécnica de Viana do Castelo, 4900-314 Viana do Castelo, Portugal

**Keywords:** institutionalised older adults, long-term care facilities, symptom burden, mental health, palliative care needs, integrated palliative care outcome scale, multidimensional assessment

## Abstract

**Background/Objectives**: Population ageing has increased the number of institutionalised older adults with multimorbidity, frailty, a high symptom burden, and complex care needs. However, data on symptom burden in residential care facilities remains limited. This study aimed to characterise the epidemiological profile of institutionalised older adults and examine the associations between symptom burden assessed using the Integrated Palliative Care Outcome Scale (IPOS) and sociodemographic characteristics, functional status, multimorbidity, depressive symptoms, loneliness, and quality of life. **Methods**: A cross-sectional correlational study was conducted between March and October 2023 in 19 residential care facilities in Portugal. Residents with preserved cognitive capacity according to education-adjusted MMSE cut-off scores completed validated instruments assessing symptom burden, functional status, depressive symptoms, loneliness, and quality of life. **Results**: The final sample comprised 306 institutionalised older adults (mean age: 85.1 years; 68.6% women). Participants had a moderate symptom burden (mean IPOS score: 18.0 ± 14.0). Depressive symptoms showed the most consistent associations with symptom burden across IPOS outcomes, while functional status showed domain-specific associations. Other associations were less robust after correction for multiple testing. Multimorbidity was not independently associated with IPOS score. **Conclusions**: These findings highlight the importance of comprehensive multidimensional assessment and support the integration of palliative care principles into long-term residential care to better address the complex needs of institutionalised older adults.

## 1. Introduction

One of the most significant structural changes in contemporary societies is population ageing. Advances in science, socio-economic conditions and technological development have resulted in an increase in the number of older adults living with multimorbidity, frailty, and progressive functional decline [1,2,3]. This clinical profile is associated with highly complex care needs and a symptom burden that is often underestimated, particularly in the advanced stages of chronic diseases [4,5]. This demographic transition poses challenges for health systems and long-term care services, as well as for the sustainability of social care provision.

It is estimated that over 20% of the European population is aged 65 years or over, with a significant proportion being 80 years or above [1]. Evidence from international organisations such as the World Health Organization (WHO) highlights the growing prevalence of frailty, multimorbidity and functional dependence in this population, reinforcing the need for adequate long-term care responses [1].

The increasing dependency ratio is expected to put significant pressure on long-term care systems, particularly residential care facilities, in the coming decades. Despite their growing importance, the quality of care provided in these settings varies considerably depending on national policies and the availability of resources. In some contexts, care is often provided by professionals with limited training in geriatric and palliative care, and staffing and resource constraints may compromise the timely identification of complex needs and the adequacy of care provided [6,7].

Many older adults living in residential care facilities present with multiple chronic conditions, cognitive impairment and limitations in activities of daily living. The identification of physical, emotional and social problems is not always integrated into routine care, which may lead to interventions that are not aligned with residents’ needs [7]. Previous studies in Portugal have highlighted the high prevalence of functional limitations, cognitive impairment, depressive symptoms and reduced quality of life among older adults in residential care settings [8,9,10].

Palliative care is a person-centred approach aimed at preventing and relieving suffering through the identification, comprehensive assessment and management of physical, psychological, social and spiritual needs [11]. This approach is particularly relevant for populations with complex chronic conditions and high symptom burden, such as institutionalised older adults [7].

Recent studies suggest that early integration of palliative care supports the identification and management of symptoms and multidimensional concerns among individuals with chronic and progressive conditions [12]. Evidence suggests that palliative care interventions may contribute to reducing symptom burden and improving quality of life [12]. However, palliative care needs in residential care facilities remain underexplored [7].

The use of patient-reported outcome measures (PROMs) has been recognised as an important strategy for supporting comprehensive assessment in palliative care [13,14]. These instruments capture patients’ perceptions of their symptoms, concerns, and overall health status, complementing traditional clinical assessments and supporting communication and clinical decision-making [14]. Evidence also suggests that the systematic use of PROMs may contribute to improved symptom assessment and patient-centred care [14,15].

The Integrated Palliative Care Outcome Scale (IPOS) is a validated multidimensional instrument designed to assess symptom burden and psychosocial concerns in individuals with advanced illness. It provides a comprehensive evaluation of patients’ experiences and supports clinical decision-making in palliative care contexts [13,16,17,18].

Despite its recognised potential, the systematic use of instruments such as IPOS in residential care facilities remains limited [7,17]. The implementation of structured multidimensional assessment instruments, such as the IPOS, may support the systematic assessment of symptom burden and facilitate the identification of complex needs among institutionalised older adults [7,17].

Given the multifactorial nature of vulnerability in this population, it is essential to analyse symptom burden in conjunction with sociodemographic characteristics, functional status, multimorbidity, depressive symptoms, loneliness and quality of life. However, evidence examining the relationship between these factors and multidimensional symptom burden among institutionalised older adults remains limited. In particular, evidence integrating older adults’ own reports of multidimensional symptom burden with functional, psychological, social and quality-of-life indicators remains limited in residential care settings [7].

In this context, the present study aimed to characterise the epidemiological profile of institutionalised older adults in southern Portugal and to examine the associations of symptom burden and multidimensional concerns with sociodemographic characteristics, functional status, multimorbidity, depressive symptoms, loneliness and quality of life.

## 2. Materials and Methods

### 2.1. Study Design and Participants

A cross-sectional correlational study was conducted between March and October 2023 in residential care facilities for older adults located in southern Portugal. These facilities provide long-term residential care for older adults with varying levels of dependency, multimorbidity, and functional and clinical complexity.

Data were collected and entered into the MIAP platform throughout this period, with each participant assessed at a single time point. Data completeness was monitored during data collection, and participating institutions were contacted when necessary to reinforce data completion procedures.

A total of 32 residential care facilities were invited to participate in the study. One institution declined participation and 12 were excluded due to incomplete institutional data. Consequently, 19 residential care facilities were included in the study. A non-probabilistic sampling strategy was used, with participating residential care facilities constituting a convenience sample. Within these facilities, all eligible residents were considered for inclusion.

Participants were eligible if they were aged 65 years or older and resided in one of the participating facilities. Written informed consent was obtained from all participants with decision-making capacity or from the institutional legal representative when participants were unable to provide informed consent themselves.

All eligible participants underwent cognitive screening using the Mini-Mental State Examination (MMSE) [19] and functional assessment using the Elderly Nursing Core Set (ENCS) [20]. Both instruments were administered by trained healthcare professionals using direct assessment and available clinical information.

Participants presenting cognitive impairment according to education-adjusted MMSE cut-off scores, severe communication difficulties, or inability to complete self-report instruments were excluded from the self-report assessment phase but remained part of the initial epidemiological characterisation of the study population.

Participants with preserved cognitive capacity subsequently completed the Patient Health Questionnaire-9 (PHQ-9) [21], UCLA Loneliness Scale [22], Integrated Palliative Outcome Scale (IPOS) [23], and WHOQOL-BREF [24].

Multimorbidity was defined as the coexistence of two or more chronic conditions. Chronic conditions were identified from residents’ clinical records and recorded in the MIAP platform using a free-text field, allowing all documented chronic conditions to be considered rather than restricting data collection to a predefined condition set. For the analysis, multimorbidity was coded as a dichotomous variable (0 = fewer than two chronic conditions; 1= two or more chronic conditions) [25].

No a priori sample size calculation was performed, as the study aimed to include all residents from the participating facilities during the data collection period. The final analytical sample therefore comprised eligible residents who were able to complete the study assessments and had complete data for the variables included in the analyses.

Initially, 1278 institutionalised residents were identified. Of these, 124 (9.7%) were excluded due to incomplete clinical records and missing assessment data, which prevented the assessment of multimorbidity and the retrieval of essential sociodemographic information. Consequently, 1154 residents were considered eligible and included in the initial assessment.

Of the 1154 eligible residents, 350 demonstrated preserved cognitive capacity according to the education-adjusted MMSE criteria and completed the self-report assessment. Of these, 44 were excluded from the analytical sample due to missing data for the study variables, resulting in a final analytical sample of 306 participants. Considering 306 participants, 19 predictor degrees of freedom, a two-sided α of 0.05, and 80% statistical power, the available sample was sufficient to detect an overall effect size of approximately f^2^ = 0.074.

Given that several study outcomes relied on self-reporting, the final analytical sample primarily represents residents with preserved cognitive capacity and the ability to complete the assessment. The exclusion of residents unable to self-report, as well as those with missing data, may therefore limit the generalisability of the findings to the broader population of institutionalised older adults.

**Figure 1:**
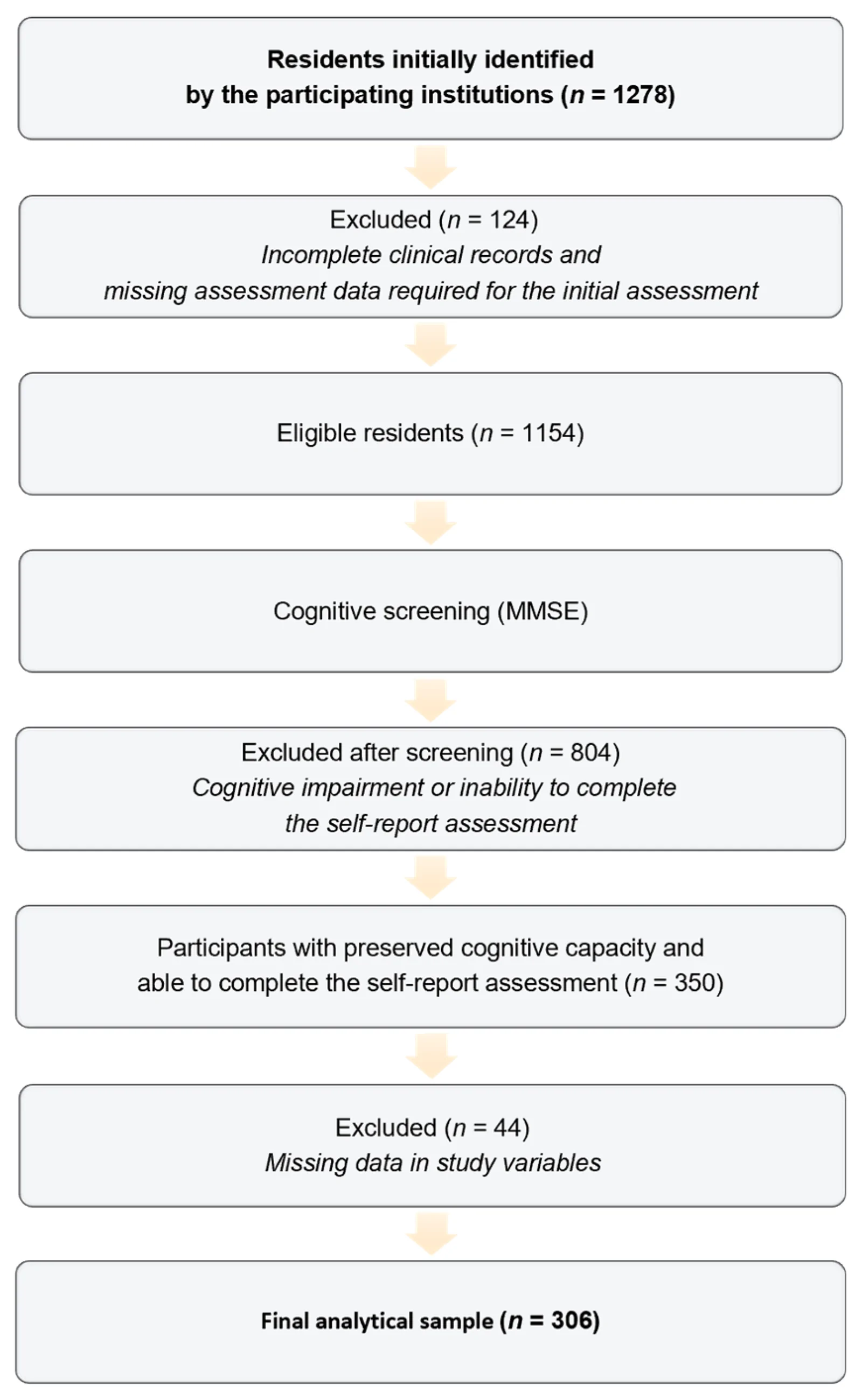
Flow diagram illustrating participant selection and inclusion in the final analytical sample.

The participant selection process is illustrated in Figure 1.

### 2.2. Sociodemographic and Clinical Variables

Sociodemographic information was collected, including sex, age, marital status, and level of education. Clinical data, including documented medical diagnoses, were extracted from institutional clinical records.

Multimorbidity was defined as the coexistence of two or more chronic conditions, based on documented medical diagnoses.

### 2.3. Instruments

All instruments used in this study were validated Portuguese versions and demonstrated adequate psychometric properties for use in the Portuguese population.

#### 2.3.1. Mini-Mental State Examination (MMSE)

Cognitive status was assessed using the Mini-Mental State Examination (MMSE), a widely used screening instrument for the global assessment of cognitive functioning, that was originally developed by Folstein and colleagues [26]. The MMSE was administered in a face-to-face format by trained healthcare professionals.

The instrument evaluates several cognitive domains, including temporal and spatial orientation, memory, attention and calculation, language, repetition, and visuoconstructive ability. Each item is scored as correct or incorrect, with total scores ranging from 0 to 30 points, where higher scores indicate better cognitive performance.

The Portuguese validated version of the MMSE was used in this study [19,27]. Education-adjusted cut-off scores were applied to screen for cognitive impairment: ≤15 points for individuals with no formal education, ≤22 points for those with less than 12 years of schooling, and ≤27 points for individuals with 12 or more years of education. Scores below these thresholds were considered indicative of cognitive impairment.

#### 2.3.2. Elderly Nursing Core Set (ENCS)

Functional status was assessed using the Elderly Nursing Core Set (ENCS), which was developed by Fonseca et al. [20] and based on the International Classification of Functioning, Disability and Health (ICF). The ENCS was completed by trained healthcare professionals based on direct assessment of participants’ functional performance. The instrument comprises 25 items rated on a five-point Likert scale and distributed across four domains: self-care, learning and mental functions, communication, and social relationships. Results are converted into percentages, allowing for classification into five levels of disability: no disability (0–4%), mild (5–24%), moderate (25–49%), severe (50–95%), and complete (96–100%). Higher scores indicate poorer functional status. The Instrument demonstrated excellent internal consistency (Cronbach’s α = 0.963) [20,28].

#### 2.3.3. Patient Health Questionnaire (PHQ-9)

Depressive symptoms were assessed using the Patient Health Questionnaire-9 (PHQ-9), a widely used instrument for depression screening in primary healthcare settings. The PHQ-9 was originally developed as a measure of depressive symptom severity [29] and has subsequently been evaluated in Portuguese-speaking populations [21,30]. The PHQ-9 was selected because the study aimed to specifically assess the presence and severity of depressive symptoms rather than provide a broader screening of emotional distress.

The nine items correspond to the DSM-IV diagnostic criteria for major depressive disorder, including depressed mood, anhedonia, sleep disturbances, fatigue, changes in appetite or weight, feelings of guilt or worthlessness, concentration difficulties, psychomotor agitation or retardation, and suicidal ideation [29].

Each item is rated on a four-point Likert scale (0–3), reflecting the frequency of symptoms over the previous two weeks. An additional item assesses the extent to which these symptoms interfere with daily functioning. Total scores range from 0 to 27, which are obtained by summing the nine symptom items. Depression severity is categorised as follows [29]: 0–4 (none), 5–9 (mild), 10–14 (moderate), 15–19 (moderately severe), and 20–27 (severe).

#### 2.3.4. UCLA Loneliness Scale (UCLA-16)

The subjective experience of loneliness was assessed using the UCLA Loneliness Scale (UCLA-16). This instrument is a widely used measure of perceived loneliness and social isolation, with established psychometric properties [31].

The Portuguese version was adapted and validated for older adults by Pocinho et al. [22]. The scale consists of 16 items with four response options and assesses perceived social isolation and relational connectedness. It demonstrates good internal consistency. Total scores range from 16 to 64, with scores above 32 indicating significant levels of loneliness [22].

#### 2.3.5. Integrated Palliative Outcome Scale (IPOS)

Symptom burden and multidimensional concerns were assessed using the Integrated Palliative Outcome Scale (IPOS). The IPOS is a patient-reported outcome measure developed to assess symptoms and other palliative care concerns from the individual’s perspective [32]. It provides a multidimensional assessment encompassing symptoms as well as psychological, social, existential, communication, information, and practical concerns [32,33].

The instrument has demonstrated validity, reliability and responsiveness in advanced illness [18] and has been culturally adapted and validated for the Portuguese population [23].

The questionnaire includes an open-ended item addressing the main problems experienced during the previous three days. Subsequent items are rated on a five-point Likert scale (0–4). The instrument covers physical symptoms as well as psychological, social and practical concerns [18,32].

For scoring purposes, 17 closed-ended items were included. The physical symptoms domain comprised 10 items, yielding a theoretical score range of 0–40; the emotional symptoms domain comprised four items, with a range of 0–16; and the communication/practical concerns domain comprised three items, with a range of 0–12. The IPOS total score was calculated as the sum of these 17 items, with a theoretical range of 0–68 [18]. Higher scores indicate greater symptom burden or greater levels of concerns. The initial open-ended question and the item concerning assistance with questionnaire completion were not included in the score [18]. For analytical purposes and to facilitate comparison between the total IPOS score and domain scores with different theoretical ranges, the total and domain-specific raw scores were linearly transformed into a 0–100 scale by dividing each observed score by its corresponding theoretical maximum and multiplying by 100. Higher scores indicate greater symptom burden or greater levels of multidimensional concerns. All IPOS results reported in the descriptive and regression analyses refer to these transformed 0–100 scores.

The patient version also includes a question regarding whether assistance was required to complete the questionnaire [18] and encourages discussion of concerns with healthcare professionals.

The instrument has been translated into multiple languages and validated across diverse healthcare contexts [17], making it a valuable tool for both clinical practice and research.

#### 2.3.6. WHOQOL-BREF

Quality of life was assessed using the WHOQOL-BREF, which was developed by the World Health Organization as a shorter version of the WHOQOL-100 instrument [24]. The WHOQOL-BREF consists of 26 items, including two global questions and four domains: physical health, psychological health, social relationships and environment. Responses are scored on a five-point Likert scale and transformed into a 0–100 scale, where higher scores indicate better perceived quality of life [24]. The instrument has been adapted and validated for the Portuguese population [34,35].

In this study, the WHOQOL-BREF was used to assess perceived quality of life, whereas the IPOS assessed symptom burden and multidimensional concerns. The two instruments were therefore considered complementary, allowing the relationship between symptom burden and perceived quality of life to be examined.

### 2.4. Data Collection Procedures

Following approval by the Ethics Committee, the directors of residential care facilities in the districts of Évora and Beja were initially contacted by telephone. Subsequently, an email containing detailed information about objectives and procedures was sent to the directors of each institution.

Prior to data collection, face-to-face training sessions were conducted at each participating facility during the three months preceding data collection. The sessions were attended by healthcare professionals selected by each institution, including nurses, psychologists, physiotherapists, social workers and occupational therapists. The study objectives, assessment instruments, and the data collection procedures were thoroughly explained to ensure consistency in data collection across facilities. A written guideline document was provided, and telephone support was available throughout the data collection period to clarify any questions that arose.

Data collection took place between March and October 2023. Participants’ responses were recorded in the Multidimensional Integrated Assessment Platform for the Elderly (MIAPe). Each participant was assigned an individual identification code to ensure anonymity and confidentiality.

Upon completion of data collection, the dataset was exported to Microsoft Excel and subsequently analysed using STATA version 15.1 (StataCorp, College Station, TX, USA).

### 2.5. Ethical Considerations

The study was conducted in accordance with the Declaration of Helsinki and approved by the Ethics Committee of the University of Évora on 11 November 2022 (reference No. 22077).

All participants received detailed information about the study. Written informed consent was obtained either directly from participants with decision-making capacity or from the institutional legal representative when participants were unable to provide informed consent themselves.

### 2.6. Statistical Analysis

Descriptive statistics were used to characterise the study sample. Continuous variables are presented as means and standard deviations, while categorical variables are reported as absolute frequencies and percentages. The distribution of total and domain-specific IPOS scores was examined using descriptive statistics and graphical representations.

Linear regression models were used to examine associations between symptom burden (IPOS total and dimensional scores) and sociodemographic, clinical, and psychological variables. To assess which factors remained associated with symptom burden after simultaneous adjustment for the other measured characteristics, multivariable linear regression models were fitted for the total IPOS score and each of its three domain-specific scores.

The multivariable models simultaneously included sex, age, marital status, educational level, multimorbidity, the four ENCS domains (self-care, learning and mental functions, communication, and relationships), the four WHOQOL-BREF domains (physical health, psychological health, social relationships, and environment), depressive symptoms, and loneliness. The overall ENCS functional profile and the two-item general quality-of-life score were not included in the multivariable models to avoid introducing redundant measures from the same instruments. Multicollinearity was assessed using variance inflation factors (VIFs), which showed no evidence of problematic multicollinearity (maximum VIF = 3.26).

Model assumptions were examined through residual-versus-fitted and Q–Q plots. Q–Q plots indicated some departure from normality in the tails, consistent with the skewed distribution of the outcomes. Heteroscedasticity was formally assessed using the Breusch–Pagan test. Evidence of heteroscedasticity was observed for the physical, emotional, and communication/practical domain models; therefore, all multivariable models were estimated using heteroscedasticity-robust standard errors. Regression coefficients (β) and corresponding 95% confidence intervals (95% CIs) are reported.

Statistical analyses were conducted using a complete-case approach for the variables included in each model. The total IPOS score was considered the main summary outcome, whereas domain-specific analyses were considered secondary and exploratory. A two-sided *p*-value < 0.05 was considered nominally statistically significant. Given the number of regression coefficients examined across the four outcomes, the Benjamini–Hochberg false discovery rate (FDR) procedure was applied across the 76 coefficient tests as a sensitivity analysis for multiple testing [36,37]; FDR-adjusted *p*-values (q-values) are also reported. All analyses were performed using STATA version 15.1 (StataCorp, College Station, TX, USA).

## 3. Results

Table 1 displays the sociodemographic, clinical and psychological characteristics of the older adults included in our study. The sample was predominantly female (68.6%), with a mean age of 85.1 years (range: 65–100). Most participants were widowed (70.3%), while 17.0% were married or living in a de facto relationship, 8.5% were single, and 4.2% were divorced or separated. Regarding educational attainment, 25.5% of participants had not attended school and were unable to read or write, 13.7% had not attended school but were literate, 58.2% had attended school but not higher education, and only 2.6% had completed higher education. Multimorbidity, defined as the presence of two or more chronic conditions, was observed in 98.0% of the sample.

In terms of functionality, mean ENCS scores indicated greater impairment in the general (mean = 2.2, SD = 0.8) and self-care domains (mean = 2.0, SD = 0.9), followed by learning and mental functions (mean = 1.8, SD = 0.7), relationships (mean = 1.6, SD = 0.6), and communication (mean = 1.4, SD = 0.6). Quality of life assessed using the WHOQOL-BREF showed lower mean scores in the physical (mean = 53.2, SD = 11.2) and psychological domains (mean = 55.3, SD = 13.3) compared with the social relationships (mean = 61.8, SD = 13.2) and environmental domains (mean = 63.6, SD = 14.2). The mean general quality of life score was 54.2 (SD = 17.3). The mean PHQ-9 score for depressive symptoms was 4.1 (SD = 4.5). Loneliness, measured using the UCLA-16 scale, had a mean score of 27.5 (SD = 9.7) (Table 1).

**Figure 2:**
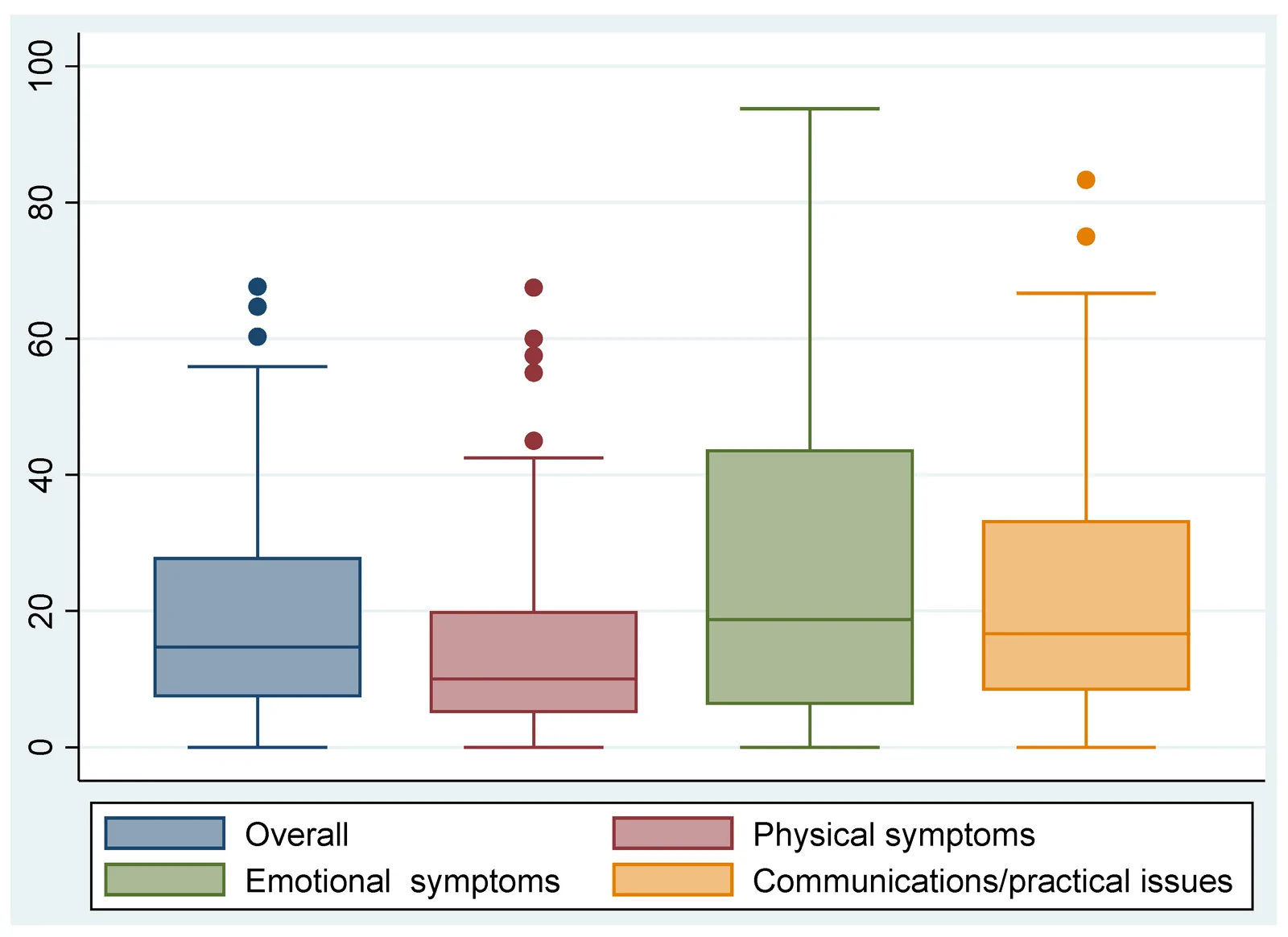
Distribution of total IPOS score and domain-specific scores. Note: Boxes represent the interquartile range, horizontal lines within boxes indicate medians, whiskers represent values within 1.5 times the interquartile range, and individual points indicate outliers. Means are reported in the accompanying text and are not represented by the horizontal lines in the boxplots. The colours of the individual points correspond to the IPOS domains identified in the figure legend.

Figure 2 presents the distribution of total IPOS and dimensional scores in the study sample. The mean total IPOS score was 18.0 (SD = 14.0; median = 14.7), indicating a moderate overall symptom burden, with scores ranging from 0 to 67.6. The distribution reflects a higher concentration of participants with lower IPOS scores and a smaller proportion reporting higher levels of palliative care needs. Regarding IPOS dimensions, the mean physical symptom score was 14.2 (SD = 12.5; median = 10.0), while the emotional symptom dimension showed the highest mean score (mean = 25.3, SD = 23.8; median = 18.8). The mean score for communication and practical issues was 20.8 (SD = 18.8; median = 16.7). The distributions were positively skewed, as reflected by means exceeding the corresponding medians and by the presence of participants with comparatively high scores.

Multicollinearity was not considered problematic, with VIF values ranging from 1.06 to 3.26. Examination of model diagnostics showed some departure from residual normality in the tails. Breusch–Pagan tests indicated evidence of heteroscedasticity for the physical symptom (*p* < 0.001), emotional symptom (*p* = 0.002), and communication/practical concern (*p* = 0.030) models, but not for the total IPOS score model (*p* = 0.098). Accordingly, heteroscedasticity-robust standard errors were used for all multivariable analyses.

Table 2 presents the simultaneously adjusted associations between participants’ sociodemographic, clinical, functional, quality-of-life, and psychological characteristics and total IPOS and domain-specific scores. Depressive symptoms showed the most consistent association with symptom burden. Each one-point increase in the PHQ-9 score was associated with a 2.12-point increase in the total IPOS score (β = 2.12; 95% CI: 1.78 to 2.45; *p* < 0.001). Depressive symptoms were also positively associated with physical symptoms (β = 1.85; 95% CI: 1.50 to 2.20; *p* < 0.001), emotional symptoms (β = 3.02; 95% CI: 2.34 to 3.69; *p* < 0.001), and communication/practical concerns (β = 1.80; 95% CI: 1.30 to 2.30; *p* < 0.001).

Functional associations varied according to the IPOS domain. Greater self-care impairment was associated with higher physical symptom scores (β = 3.37; 95% CI: 2.01 to 4.74; *p* < 0.001). Greater communication impairment was nominally associated with greater communication/practical concerns (β = 5.85; 95% CI: 1.47 to 10.23; *p* = 0.009), and greater impairment in relationships was nominally associated with higher emotional symptom scores (β = 4.38; 95% CI: 0.09 to 8.67; *p* = 0.045). An inverse association was also observed between self-care impairment and communication/practical concerns (β = −3.89; 95% CI: −6.30 to −1.49; *p* = 0.002).

None of the four WHOQOL-BREF domains was independently associated with the total IPOS score or domain-specific scores at the nominal *p* < 0.05 level after simultaneous adjustment. Loneliness was nominally associated with emotional symptoms (β = 0.29; 95% CI: 0.01 to 0.58; *p* = 0.043) and communication/practical concerns (β = 0.31; 95% CI: 0.06 to 0.57; *p* = 0.017), but not with the total IPOS or physical symptom scores. Some nominal associations were also observed for sex, marital status, and educational level (Table 2). Multimorbidity was not independently associated with any IPOS outcome.

After Benjamini–Hochberg correction for multiple testing across the 76 regression coefficient tests, depressive symptoms remained significantly associated with the total IPOS score and all three domain-specific scores (all q < 0.001). The association between self-care impairment and physical symptoms also remained significant (q < 0.001), as did the inverse association between self-care impairment and communication/practical concerns (q = 0.019). None of the remaining nominal associations remained statistically significant after FDR correction.

## 4. Discussion

The present study aimed to characterise the epidemiological profile of institutionalised older adults and to examine the associations between symptom burden and multidimensional concerns and sociodemographic characteristics, functional status, multimorbidity, depressive symptoms, loneliness and quality of life. The findings revealed a substantial burden of symptoms and concerns among this vulnerable population, highlighting the complexity of care and the relevance of comprehensive assessment approaches in residential care settings.

The study sample was characterised by advanced age, a predominance of women, and a high prevalence of multimorbidity. These findings are consistent with previous national and international studies indicating that institutionalised older adults are more likely to be female, of advanced age, and affected by multiple chronic conditions [2,3,10,20,38]. Multimorbidity is recognised as a major challenge in ageing populations due to its association with functional decline, increased dependency and greater healthcare utilisation [25,39].

In long-term care settings, multimorbidity frequently coexists with frailty, functional dependence, and polypharmacy, increasing vulnerability and complicating care delivery [2,3,39]. Previous studies involving institutionalised older adults have demonstrated strong associations between functional impairment, depressive symptoms, loneliness, cognitive decline, and reduced quality of life [8,9,10,20], reinforcing the multidimensional nature of care needs in this population. Together, these findings support the importance of integrated and person-centred approaches to assessment and care planning.

The results also indicated an overall symptom burden, as assessed by the IPOS, reflecting the presence of multidimensional concerns among institutionalised older adults. Similar findings have been reported in studies using the IPOS to assess palliative care needs, demonstrating that older adults with chronic and progressive conditions frequently experience concurrent physical, emotional and psychosocial symptoms that adversely affect their quality of life [5,12,18,40]. The findings highlight the value of multidimensional symptom assessment in residential care settings.

Regarding the second study objective, the multivariable analyses showed that the relationship between functional status and symptom burden differed according to the functional and symptom domains considered. Greater self-care impairment was associated with higher physical symptom burden after simultaneous adjustment for sociodemographic characteristics, multimorbidity, the remaining functional domains, quality of life, depressive symptoms, and loneliness. Importantly, this association remained statistically significant after correction for multiple testing. This finding is clinically plausible, as limitations in self-care may coexist with greater physical symptom burden and dependency among institutionalised older adults. Previous studies have similarly demonstrated close relationships between functional impairment, physical health, and greater care needs in this population [7,8,9,20]. Greater communication impairment was also associated, at the nominal significance level, with greater communication/practical concerns, while greater impairment in social relationships was nominally associated with higher emotional symptom scores. These associations did not remain statistically significant after FDR correction and should therefore be interpreted as exploratory. Nevertheless, the correspondence between specific functional and symptom domains supports the multidimensional nature of residents’ needs and warrants further investigation.

An unexpected inverse association was observed between self-care impairment and communication/practical concerns, and this association also remained statistically significant after FDR correction. Because higher ENCS scores indicate greater functional impairment, the direction of this coefficient suggests that, conditional on the other variables included in the model, greater self-care impairment was associated with lower communication/practical concern scores. This finding should be interpreted cautiously. The simultaneous inclusion of several related functional domains may produce conditional or suppression effects even in the absence of problematic multicollinearity [41]. Accordingly, this result should not be interpreted as indicating that greater self-care impairment is protective against communication or practical concerns, and replication in other samples is warranted.

In contrast, none of the WHOQOL-BREF domains was independently associated with the total IPOS score or scores for its domains in the multivariable models. Although symptom burden and quality of life are conceptually and clinically related, the absence of statistically significant associations after simultaneous adjustment suggests that the contribution of perceived quality of life may overlap with other dimensions included in the model, particularly psychological, social, and functional factors. Previous studies have demonstrated close relationships between greater symptom burden and poorer quality of life [5,12,13,42,43]; however, the present findings indicate that these relationships may be difficult to disentangle when multiple interrelated dimensions are considered simultaneously [41].

Depressive symptoms showed the most consistent association with symptom burden in the present study. Higher PHQ-9 scores remained strongly associated with the total IPOS score and with the scores for the physical, emotional, and communication/practical domains after simultaneous adjustment for sociodemographic characteristics, multimorbidity, functional status, quality of life, and loneliness. Moreover, these associations remained statistically significant after FDR correction for multiple testing. These findings are consistent with previous studies demonstrating that psychological distress is a major component of palliative care needs among older adults with advanced illness [5,12,44]. Studies evaluating the use of the IPOS have also identified emotional symptoms and psychosocial concerns as core dimensions of symptom burden, supporting its value for the multidimensional assessment of palliative care needs [13,17,43]. The particularly strong association between PHQ-9 scores and the emotional IPOS domain should nevertheless be interpreted in light of the conceptual overlap between depressive symptoms and some of the emotional concerns assessed by the IPOS. However, the persistence of the association across all IPOS domains, including physical symptoms and communication/practical concerns, suggests that depressive symptomatology may be related to a broader multidimensional symptom experience rather than exclusively to the emotional component of the IPOS [5,12,44].

Loneliness was positively associated with the emotional and communication/practical IPOS domains at the nominal significance level, whereas no statistically significant association was observed with the total IPOS or physical symptom scores. However, the domain-specific associations did not remain statistically significant after FDR correction and should therefore be considered exploratory. Their direction is nevertheless consistent with the potential relationship between perceived social disconnection and emotional or interpersonal concerns in later life [45,46]. Further studies are needed to determine whether loneliness contributes to specific dimensions of symptom burden independently of depressive symptoms and other social and functional factors.

Sociodemographic factors also showed nominal associations with specific IPOS outcomes. Participants who had not attended school but were able to read and write had lower total, physical, and emotional IPOS scores than those who had not attended school and were unable to read or write. Widowhood was nominally associated with greater emotional symptom burden, and women had nominally higher physical symptom scores than men. However, none of these associations remained statistically significant after FDR correction. They should therefore be interpreted cautiously and regarded as hypothesis-generating rather than as robust evidence of independent sociodemographic effects. Although previous evidence supports relationships between education, social circumstances, psychological vulnerability, and health outcomes among older adults [39,40,41], further studies with larger and more balanced subgroups are needed to clarify these associations.

Multimorbidity was not independently associated with the total IPOS score or any of its domains in the multivariable analyses. This finding should, however, be interpreted with considerable caution. Among participants, more than 98% had two or more chronic conditions. This extreme imbalance substantially reduced both the discriminative capacity of the variable and the statistical power and precision of the comparison, as reflected in the wide confidence intervals. Consequently, the absence of statistically significant associations should not be interpreted as evidence that multimorbidity is unrelated to symptom burden in institutionalised older adults [47].

Overall, these findings highlight the multidimensional nature of symptom burden among institutionalised older adults while also showing that the strength of associations differs considerably across the factors and domains examined. Depressive symptoms showed the most consistent relationship with symptom burden and remained associated with the total IPOS score and all three domain-specific scores after correction for multiple testing. Functional status showed more domain-specific relationships, with greater self-care impairment associated with greater physical symptom burden. An unexpected inverse association between self-care impairment and communication/practical concerns also remained statistically significant and requires cautious interpretation. By contrast, the WHOQOL-BREF domains, multimorbidity, loneliness, and sociodemographic characteristics did not show robust associations after FDR correction. These findings support comprehensive multidimensional assessment while also emphasising the need to distinguish robust associations from exploratory domain-specific findings.

This study contributes to the growing body of evidence on the multidimensional needs of institutionalised older adults by providing a comprehensive epidemiological characterisation of symptom burden and its associated factors in residential care settings. Evidence specifically focusing on institutionalised older adults living in long-term residential facilities remains limited [5,12,17,42,48].

By simultaneously integrating measures of functional status, quality of life, depressive symptoms, loneliness, and multidimensional symptom burden, the present study provides further insight into the complex interplay between physical, psychological, and social factors associated with symptom burden among institutionalised older adults.

Furthermore, this study extends the available evidence on the application of the Portuguese version of the Integrated Palliative Care Outcome Scale (IPOS) among institutionalised older adults, a population in which its use has been less extensively investigated [23].

These findings also have important implications for health and social care policy. Residential care facilities increasingly support older adults with complex clinical profiles characterised by advanced age, multimorbidity, and functional dependence. However, care models in these settings are often organised primarily around social support rather than integrated healthcare provision [6,17,49,50].

The results of this study highlight the need to strengthen integration between health and social care services in long-term residential settings, particularly through systematic multidimensional assessment of residents’ needs.

The routine use of structured assessment tools such as the Integrated Palliative Care Outcome Scale (IPOS) may support the systematic identification of symptom burden and psychosocial concerns, thereby informing care planning and multidisciplinary decision-making.

Integrating palliative care principles into residential care settings may therefore represent an important strategy for promoting comprehensive assessment, coordinated care planning, and person-centred care for institutionalised older adults with complex needs.

## 5. Strengths and Limitations

This study has several strengths. It included a multicentre sample of institutionalised older adults from 19 residential care facilities, providing a comprehensive overview of symptom burden and its associated factors in this population. A multidimensional assessment approach was adopted, incorporating validated instruments to evaluate functional status, quality of life, depressive symptoms, loneliness, and symptom burden. In addition, this study extends the available evidence on the application of the Portuguese version of the Integrated Palliative Care Outcome Scale (IPOS) among institutionalised older adults, a population in which its use has been less extensively investigated, although the instrument has previously been validated for Portuguese adults with advanced illness in different healthcare settings [23].

However, some limitations should be acknowledged. The cross-sectional design precludes the establishment of causal relationships between variables. In addition, the use of a convenience sampling strategy may limit the generalisability of the findings. The very high prevalence of multimorbidity resulted in a small reference group without multimorbidity, limiting the statistical power and precision of comparisons and requiring cautious interpretation of the absence of statistically significant associations. Finally, 804 (69.7%) of the 1154 eligible residents were excluded from the self-report phase due to cognitive impairment or inability to complete the self-report assessment, which may have led to the underrepresentation of individuals with more severe cognitive and functional impairment and limits the generalisability of the findings to the broader population of institutionalised older adults. Future studies should specifically address the needs of residents with cognitive impairment using assessment approaches appropriate to their cognitive and communication abilities. Additionally, data collection took place between March and October 2023, spanning different seasons. Potential seasonal or other time-related effects were not specifically assessed or controlled for and should therefore be considered when interpreting the findings.

## 6. Conclusions

This study shows that institutionalised older adults experience a substantial multidimensional symptom burden. Depressive symptom showed the most consistent association with symptom burden, while functional status showed domain-specifical relationships. Associations involving loneliness, quality of life, sociodemographic characteristics were less robust after adjustment for multiple testing, highlighting the complex interplay between physical, psychological and social needs in this population.

These findings reinforce the importance of comprehensive multidimensional assessment that incorporates the older person’s own report of symptoms and concerns to identify the complex needs of institutionalised older adults. The use of structured assessment instruments, such as the Integrated Palliative Care Outcome Scale (IPOS), may support the systematic assessment of symptom and related concerns in residential care settings. The findings also support the integration of palliative care principles into long-term residential care through comprehensive assessment and coordinated care planning that addresses physical, psychological, social, and spiritual needs. Particular attention should be given to functional status, psychological well-being, social relationships, and quality of life when assessing needs and planning care for institutionalised older adults.

## Figures and Tables

**Table 1:** Sociodemographic, clinical and psychological characteristics of the participants.

	*n* = 306
**Sex, *n* (%)**	
Male	96 (31.4)
Female	210 (68.6)
**Age (years)**	
Mean (min–max)	85.1 (65–100)
**Marital status, *n* (%)**	
Single	26 (8.5)
Married/in a de facto relationship	52 (17.0)
Widower	215 (70.3)
Divorced/separated	13 (4.2)
**Education, *n* (%)**	
Did not attend school and cannot read or write	78 (25.5)
Did not attend school, but can read and write	42 (13.7)
Attended school but not higher education	178 (58.2)
Attended higher education	8 (2.6)
**Multimorbidity ^¶^, *n* (%)**	
No	6 (2.50)
Yes	299 (98.0)
**Functionality (ENCS)** ^¥^**, mean (SD)**	
Self-care	2.0 (0.9)
Learning and mental functions	1.8 (0.7)
Communication	1.4 (0.6)
Relationships	1.6 (0.6)
General	2.2 (0.8)
**Quality of life (WHOQOL-BREF)** ^§^**, mean (SD)**	
Physical domain	53.2 (11.2)
Psychological domain	55.3 (13.3)
Domain of social relationships	61.8 (13.2)
Environmental domain	63.6 (14.2)
General quality of life	54.2 (17.3)
**Depressive symptoms (PHQ9)** ^£^**, mean (SD)**	
Overall score	4.1 (4.5)
**Loneliness (UCLA-16)** ^¤^**, mean (SD)**	
Overall score	27.5 (9.7)

^¶^ Diagnosis of two or more chronic diseases. ^¥^ Elderly Nursing Core Set (ENCS; range: 1–5). ^§^ World Health Organization Quality of Life–BREF (WHOQOL-BREF; range: 0–100). ^£^ 9-Item Patient Health Questionnaire (PHQ-9; range: 0–27). ^¤^ The Loneliness Scale for Portuguese Elderly (UCLA; range: 16–64).

**Table 2:** Multivariable associations between participants’ sociodemographic, clinical, functional, quality-of-life, and psychological characteristics and total IPOS and domain-specific scores.

	IPOS Adjusted β (95% CI); *p*; q			
	Overall	Physical Symptoms	Emotional Symptoms	Communications/ Practical Issues
**Sex**				
Female vs. male	1.97 (−0.24 to 4.18); *p* = 0.080; q = 0.320	2.26 (0.06 to 4.45); *p* = 0.044; q = 0.230	3.02 (−1.28 to 7.33); *p* = 0.168; q = 0.466	−0.38 (−4.38 to 3.62); *p* = 0.852; q = 0.938
**Age (years)**	0.01 (−0.15 to 0.17); *p* = 0.902; q = 0.961	−0.03 (−0.17 to 0.10); *p* = 0.615; q = 0.779	0.13 (−0.22 to 0.47); *p* = 0.477; q = 0.659	0.0 (−0.31 to 0.32); 1.0 *p* = 0.980; q = 0.980
**Marital status**				
Married/in a de facto relationship vs. Single	2.18 (−1.45 to 5.81); *p* = 0.239; q = 0.553	1.79 (−1.57 to 5.16); *p* = 0.295; q = 0.571	5.00 (−2.17 to 12.16); *p* = 0.172; q = 0.466	−0.29 (−6.76 to 6.18); *p* = 0.930; q = 0.968
Widowed vs. Single	2.61 (−0.71 to 5.93); *p* = 0.123; q = 0.410	1.10 (−2.00 to 4.20); *p* = 0.488; q = 0.662	6.25 (0.34 to 12.15); *p* = 0.038; q = 0.230	2.82 (−3.19 to 8.83); *p* = 0.358; q = 0.579
Divorced/separated vs. Single	4.54 (−1.41 to 10.49); *p* = 0.135; q = 0.410	2.64 (−3.44 to 8.72); *p* = 0.394; q = 0.599	7.40 (−5.34 to 20.15); *p* = 0.255; q = 0.553	7.04 (−4.17 to 18.25); *p* = 0.218; q = 0.535
**Education**				
Did not attend school, but can read and write vs. Did not attend school and cannot read or write	−4.43 (−7.97 to −0.89); *p* = 0.014; q = 0.125	−3.85 (−6.94 to −0.75); *p* = 0.015; q = 0.125	−8.80 (−16.15 to −1.45); *p* = 0.019; q = 0.131	−0.54 (−6.67 to 5.59); *p* = 0.864; q = 0.938
Attended school but not higher education vs. Did not attend school and cannot read or write	−1.13 (−4.04 to 1.78); *p* = 0.446; q = 0.640	−1.76 (−4.85 to 1.32); *p* = 0.263; q = 0.553	−0.76 (−6.29 to 4.78); *p* = 0.789; q = 0.909	0.49 (−4.09 to 5.06); *p* = 0.835; q = 0.933
Attended higher education vs. Did not attend school and cannot read or write	−4.11 (−9.40 to 1.18); *p* = 0.128; q = 0.410	−4.21 (−8.81 to 0.40); *p* = 0.073; q = 0.320	−4.79 (−13.85 to 4.28); *p* = 0.301; q = 0.571	−2.88 (−11.37 to 5.61); *p* = 0.506; q = 0.674
**Multimorbidity ^¶^**				
Yes vs. no	2.74 (−4.74 to 10.21); *p* = 0.473; q = 0.659	2.68 (−2.12 to 7.49); *p* = 0.274; q = 0.553	7.11 (−2.20 to 16.43); *p* = 0.134; q = 0.410	−2.93 (−22.56 to 16.70); *p* = 0.770; q = 0.900
**Functionality**				
Self-care	1.03 (−0.30 to 2.36); *p* = 0.131; q = 0.410	3.37 (2.01 to 4.74); *p* < 0.001; q < 0.001	−1.15 (−4.00 to 1.69); *p* = 0.428; q = 0.636	−3.89 (−6.30 to −1.49); *p* = 0.002; q = 0.019
Learning and mental functions	−0.95 (−3.11 to 1.20); *p* = 0.385; q = 0.598	−0.36 (−2.28 to 1.57); *p* = 0.716; q = 0.864	−2.10 (−6.53 to 2.32); *p* = 0.351; q = 0.579	−1.41 (−4.96 to 2.14); *p* = 0.435; q = 0.636
Communication	1.10 (−1.39 to 3.60); *p* = 0.386; q = 0.598	−0.76 (−3.18 to 1.66); *p* = 0.537; q = 0.700	2.21 (−2.32 to 6.74); *p* = 0.339; q = 0.572	5.85 (1.47 to 10.23); *p* = 0.009; q = 0.096
Relationships	1.25 (−0.87 to 3.36); *p* = 0.249; q = 0.553	0.96 (−1.00 to 2.92); *p* = 0.336; q = 0.572	4.38 (0.09 to 8.67); *p* = 0.045; q = 0.230	−1.98 (−5.56 to 1.59); *p* = 0.276; q = 0.553
**Quality of life (WHOQOL-BREF)**				
Physical domain	−0.11 (−0.22 to 0.01); *p* = 0.079; q = 0.320	−0.06 (−0.17 to 0.06); *p* = 0.318; q = 0.572	−0.15 (−0.40 to 0.10); *p* = 0.241; q = 0.553	−0.21 (−0.42 to 0.01); *p* = 0.064; q = 0.302
Psychological domain	−0.00 (−0.12 to 0.11); *p* = 0.943; q = 0.968	0.06 (−0.05 to 0.17); *p* = 0.310; q = 0.572	−0.07 (−0.31 to 0.16); *p* = 0.543; q = 0.700	−0.12 (−0.30 to 0.07); *p* = 0.218; q = 0.535
Domain of social relationships	0.08 (−0.04 to 0.19); *p* = 0.196; q = 0.514	0.07 (−0.03 to 0.17); *p* = 0.158; q = 0.461	0.11 (−0.11 to 0.33); *p* = 0.329; q = 0.572	0.04 (−0.16 to 0.24); *p* = 0.700; q = 0.858
Environmental domain	0.02 (−0.09 to 0.13); *p* = 0.728; q = 0.864	0.00 (−0.09 to 0.10); *p* = 0.963; q = 0.975	0.06 (−0.18 to 0.29); *p* = 0.629; q = 0.784	0.02 (−0.18 to 0.23); *p* = 0.821; q = 0.931
**Depressive symptoms (PHQ-9)**	2.12 (1.78 to 2.45); *p* < 0.001; q < 0.001	1.85 (1.50 to 2.20); *p* < 0.001; q < 0.001	3.02 (2.34 to 3.69); *p* < 0.001; q < 0.001	1.80 (1.30 to 2.30); *p* < 0.001; q < 0.001
**Loneliness (UCLA-16)**	0.13 (−0.02 to 0.28); *p* = 0.099; q = 0.376	0.01 (−0.13 to 0.14); *p* = 0.910; q = 0.961	0.29 (0.01 to 0.58); *p* = 0.043; q = 0.230	0.31 (0.06 to 0.57); *p* = 0.017; q = 0.127
**R^2^**	0.665	0.632	0.509	0.367
**Adjusted R^2^**	0.642	0.607	0.475	0.324

Note: **^¶^** Multimorbidity was defined as the coexistence of two or more chronic conditions. Values are multivariable linear regression coefficients (β) with 95% confidence intervals estimated using heteroscedasticity-robust standard errors. All variables shown were entered simultaneously in each model. Higher ENCS scores indicate greater functional impairment; higher WHOQOL-BREF scores indicate better quality of life; higher PHQ-9 and UCLA-16 scores indicate greater depressive symptoms and loneliness, respectively. *p*-values are nominal two-sided *p*-values; q-values are Benjamini–Hochberg false discovery rate-adjusted *p*-values calculated across the 76 regression coefficient tests (excluding intercepts). Reference categories were male sex, single marital status, no formal schooling and inability to read or write, and absence of multimorbidity. CI, confidence interval; ENCS, Elderly Nursing Core Set; PHQ-9, Patient Health Questionnaire-9; UCLA-16, UCLA Loneliness Scale.

## Data Availability

The dataset contains sensitive information obtained from institutionalised older adults, including clinical, functional, and psychological data. Although the data have been anonymised, they cannot be made publicly available due to ethical and privacy considerations, in accordance with the approval granted by the Ethics Committee of the University of Évora and the informed consent obtained from participants and/or their legal representatives. Therefore, the data are available from the corresponding author upon reasonable request for scientific purposes, provided that the request complies with the applicable ethical and data protection requirements.
